# Practitioner/Practice- and Patient-Based Factors Contributing to Dental Practitioner Treatment Recommendations for Patients with Pain-Related TMDs: Findings from the National Dental PBRN

**DOI:** 10.11607/ofph.3263

**Published:** 2023-11-17

**Authors:** Joseph L. Riley III, D. Brad Rindal, Ana Miriam Velly, Gary C. Anderson, Kimberly S. Johnson, Gregg H. Gilbert, Eric L. Schiffman, National Dental Practice-Based Research Network Collaborative Group*

**Affiliations:** Department of Community Dentistry and Behavioral Science; College of Dentistry, University of Florida; HealthPartners Institute; Faculty of Dental Medicine and Oral Sciences; McGill University;; Network for Canadian Dental Research; Department of Developmental and Surgical Sciences; School of Dentistry; University of Minnesota; HealthPartners Institute; Department of Clinical and Community Sciences; School of Dentistry; University of Alabama at Birmingham; Department of Diagnostic and Biological Sciences; School of Dentistry, University of Minnesota; *The National Dental PBRN Collaborative Group comprises practitioners, faculty, and staff investigators who contributed to the network activity. A list of these persons is available at: http://www.nationaldentalpbrn.org/collaborative-group.php

**Keywords:** temporomandibular disorders, National Dental Practice-Based Research Network, intraoral appliance, self-care, treatment recommendations

## Abstract

**Aims:**

To document National Dental Practice-Based Research Network (PBRN) practitioner treatment recommendations for patients with painful temporomandibular disorders (TMDs) and to identify practitioner/practice- and patient-related factors contributing to treatment recommendations made at the initial clinical visit.

**Methods:**

This prospective single-sample cohort study formed groups based on treatment recommendations made by 185 dental practitioners who treated 1,901 patients with painful TMDs. At the baseline visit, which this article describes, practitioners provided patients with their diagnoses and a treatment plan and then completed a comprehensive questionnaire.

**Results:**

Self-care, an intraoral appliance, medication, and practitioner-recommended jaw exercises were the most frequently recommended treatments. Practitioners recommended multiple treatments to most patients. TMD signs, symptoms, and diagnoses were primary considerations in treatment planning, but the practitioner’s expectations for improvement were only significant for intraoral appliances and self-care. Female practitioners and those with expertise in TMDs more frequently recommended patient-directed and multidisciplinary treatments compared to their counterparts.

**Conclusions:**

Practitioners used a wide range of treatments for patients with few consistent patterns. The propensity to use TMD signs, symptoms, and diagnoses when making treatment recommendations suggests a tendency to conceptualize patients using the biomedical model. Infrequent referral to nondental providers suggests a lack of availability of these providers, a misunderstanding of the complexity of TMDs, and/or discomfort with assessment of psychosocial factors. Implications include the need for comprehensive training in the assessment and management of TMD patients during dental school and participation in TMD continuing education courses following evidence-based guidelines. *Int J Oral Facial Pain Headache 2023;37:191–202. doi: 10.11607/ofph.3263*

Temporomandibular disorders (TMDs) are commonly occurring musculoskeletal and neuromuscular conditions characterized by pain in the masticatory muscles and temporomandibular joints (TMJs), headache, limitations in mandibular movement, and joint sounds while opening or closing.^1^ In a prospective cohort study, the incidence of first onset of pain-related TMDs was approximately 4% per year, with an increased onset among women.^2^ Over time, pain-related TMDs can have a substantial negative impact on daily life, particularly in the areas of psychologic distress, physical disability, and functional limitations.^3,4^

Diagnosis and treatment of TMDs are within the purview of dentistry, with most patients first consulting a general dental practitioner.^5^ TMD treatment options include intraoral appliances, behavioral modification, jaw exercises, psychologic counselling, and pharmacologic treatments as first-choice therapies.^1,6–8^ In a National Dental Practice-Based Research Network survey, Velly et al^5^ reported that dental practitioners treated an average of three TMD patients per month. These practitioners often used more than one treatment, and the most common combination was self-care and intraoral appliances.

Factors associated with treatment selection can be categorized as practitioner/practice-related and patient-related factors. The belief that pain medication will be successful when treating pain is associated with increased use among both nurses^9^ and physicians.^10^ Female health care providers show greater attention to preventive aspects of patient care and often make more conservative treatment recommendations than their male counterparts.^11,12^ Results from the Network study^5^ indicate that practitioner perceptions of patient acceptability, anticipated compliance, cost, and sideeffects are factors associated with different TMD treatments.

Objectives of this Network prospective single-sample cohort study were to identify factors contributing to TMD treatment recommendations and to describe observed changes in pain intensity and jaw function at 1-, 3-, and 6-month follow-ups. In this report, we identify practitioner/practice- and patient-related factors contributing to dental practitioner treatment recommendations at the initial clinical visit.

## Materials and Methods

### Overview

This manuscript reports baseline data from a 6-month prospective cohort study collected in the offices of dental practitioners who were members of the Network. The Network is composed of six regions spanning the USA.^13^ Each Network IRB reviewed and approved the study protocol. A detailed overview of the study methods, including a report of practitioner treatment recommendations, has previously been published.^14^

### Study Participants and Recruitment

#### Practitioner enrollment

To participate in the study, practitioners first completed the study and protocol training. The practitioners received compensation for their participation.

#### Patient enrollment

Each practitioner was asked to recruit a target of 11 consecutive eligible consenting patients with a maximum of 20 patients. All participating patients provided informed consent. Network practitioners were instructed to recruit consecutive eligible patients with acute or chronic painful TMDs aged ≥ 18 years (except in Nebraska, where the consent age is ≥ 19 years).

### Data Collection

Following a diagnosis of painful TMDs,^15^ practitioners completed the Initial Doctor Questionnaire, which included the patient’s chief complaint, TMD diagnosis, treatment recommendations, and anticipated treatment result and difficulties. This is an observational study, and, as such, we did not change what the practitioners were currently doing, including with regard to diagnosing or treating patients. The specific treatments recommended were assessed using a checklist of common treatment options. Other potential predictors for treatment recommendations were assessed using a practitioner demographics questionnaire and descriptors of the practice. Patient sociodemographic factors were also recorded.

Practitioner treatment recommendations or referrals have already been reported.^14^ This report examines the following categories of treatment modalities: *(1)* intraoral appliance, *(2)* pain medications, *(3)* self-care, *(4)* practitioner-recommended jaw exercises, and *(5)* referral to providers for physical/biomedical treatment, occlusal stabilization, and psychologic counseling.

### Statistical Models

This single-sample study formed seven prediction models based on whether the practitioner recommended the following treatments:

1. An intraoral appliance (Table 1)
2. One or more medications (Table 2)
3. Self-care (Table 3)
4. Practitioner-recommended jaw exercises (Table 3)
5. Referral to other provider for physical/biomedical treatments (Table 4)
6. Referral for occlusal stabilization treatment (Table 4)
7. Referral for psychologic treatment (Table 4)

**Table 1:** Coefficients for GEEs of Practitioner/Practice- and Patient-Related Factors Predicting Intraoral Appliance Recommendation

Predictors	Category	B (SE)	OR (95% CI)	*P* *
Female sex (patient)	Males	0	1 (reference)	< .001
	Females	.506 (0.150)	1.66 (1.24, 2.23)	
Insurance coverage for TMD treatment	No	0	1 (reference)	
	Yes	.519 (0.166)	1.68 (1.21, 2.33)	.002
Years since dental school graduation	Interval	.019 (0.006)	1.02 (1.01, 1.03)	< .001
Number of TMD patients seen in the past month	Interval	.034 (0.007)	1.04 (1.02, 1.05)	< .001
In full-time practice	Part-time	0	1 (reference)	
	Full-time	.645 (0.146)	1.91 (1.43, 2.54)	< .001
Diagnosis of myalgia	No	0	1 (reference)	.005
	Yes	.369 (0.131)	1.45 (1.12, 1.87)	
Diagnosis of headache related to TMDs	No	0	1 (reference)	< .001
	Yes	.753 (0.127)	2.12 (1.66, 2.72)	
Jaw stiffness or fatigue	No	0	1 (reference)	< .001
	Yes	.466 (0.134)	1.59 (1.23, 2.07)	
Practitioner’s expectation for improvement in the patient’s jaw function	Ordinal	.184 (0.032)	1.20 (1.13, 1.28)	< .001

*Values are adjusted for variables in the final model.

Using listwise deletion, the final model for an intraoral appliance included 1,772 patients. Explanatory group coded = 1. Model fit: QICC baseline = 1,980.15, QICC final = 1,726.088.

**Table 2:** Coefficients for GEEs of Practitioner/Practice- and Patient-Related Factors Predicting Recommendation for Medication(s)

	Category	B (SE)	OR (95% CI)	*P* *
Expertise in TMDs and orofacial pain	No	0	1 (reference)	< .001
	Yes	–1.024 (0.103)	0.36 (0.29, 0.44)	
Diagnosis of myofascial pain with referral	No	0	1 (reference)	< .001
	Yes	0.438 (0.106)	1.55 (1.26, 1.91)	
Ear pain	No	0	1 (reference)	.001
	Yes	0.360 (0.110)	1.43 (1.16, 1.78)	
Jaw stiffness or fatigue	No	0	1 (reference)	< .001
	Yes	0.404 (0.114)	1.50 (1.20, 1.87)	
How well the patient understood the treatment recommendation	Ordinal	–0.146 (0.034)	0.86 (0.81, 0.93)	< .001

*Values are adjusted for variables in the final model.

Using listwise deletion, the final model for a medication recommendation included 1,829 patients. Model fit: QICC baseline = 2,532.342, QICC final = 2,351.523.

**Table 3:** Coefficients for GEEs of Practitioner/Practice- and Patient-Related Factors Predicting Self-Care Behaviors and Jaw Exercises

	Category	B (SE)	OR (95% CI)	*P* *
**Self-care** **^a^**				
Female sex (practitioner)	Male	0	1 (reference)	< .001
	Female	1.536 (0.250)	4.64 (2.85,7.57)	
Years since dental school graduation	Interval	0.021 (0.007)	1.02 (1.01,1.04)	.005
Expertise in TMDs and orofacial pain	No	0	1 (reference)	.006
	Yes	0.564 (0.206)	1.76 (1.17, 2.63)	
Number of TMD patients seen in the past month	Interval	–0.023 (0.006)	0.98 (0.97, 0.99)	< .001
Number of dental patients seen per week	Interval	0.015 (0.004)	1.02 (1.01, 1.02	< .001
Jaw pain	No	0	1 (reference)	.004
	Yes	0.755 (0.261)	2.13 (1.28,3.55)	
Improving patient’s ability to use their jaw	Ordinal	0.126 (0.049)	1.13 (1.03,1.25)	.009
**Jaw exercises** **^b^**				
Female sex (practitioner)	Male	0	1 (reference)	< .001
	Female	0.586 (0.104)	1.80 (1.47, 2.20)	
Diagnosis of myalgia	No	0	1 (reference)	< .001
	Yes	0.593 (0.118)	1.81 (1.44, 2.28)	
Diagnosis of myofascial pain with referral	No	0	1 (reference)	< .001
	Yes	0.648 (0.103)	1.91 (1.56, 2.34)	
TMJ noises	No	0	1 (reference)	< .001
	Yes	0.527 (0.106)	1.69 (1.38, 2.09)	
Ear pain	No	0	1 (reference)	< .001
	Yes	0.444 (0.112)	1.56 (1.25, 1.94)	
Limited jaw opening	No	0	1 (reference)	< .001
	Yes	0.433 (0.108)	1.54 (1.25, 1.90)	
Easy for patient to follow treatment recommendations	Ordinal	–0.101 (0.027)	0.90 (0.86, 0.95)	< .001

*Values are adjusted for variables in the final model.

^a^Using listwise deletion, the final model for self-care included 1,691 patients. Model fit: QICC baseline = 1,147.274, QICC final = 981.418.

^b^Using listwise deletion, the final model for active home exercises included 1,817 patients. Model fit: QICC baseline = 2,548.319, QICC final = 2,316.450.

**Table 4:** Coefficients for GEEs of Practitioner/Practice- and Patient-Related Factors Predicting Referral to an Allied Health Care Provider for Selected Specialized Treatments

	Category	B (SE)	OR (95% CI)	*P* *
**Physical/biomedical treatments** **^a^**				
Female sex (practitioner)	Male	0	1 (reference)	< .001
	Female	0.714 (0.116)	2.04 (1.63, 2.56)	
Expertise in TMDs and orofacial pain	No	0	1 (reference)	< .001
	Yes	0.753 (0.129)	2.12 (1.65, 2.73)	
Number of TMD patients seen in the past month	Interval	0.008 (0.003)	1.01 (1.01, 1.02	.001
Number of dental patients seen per week	Interval	–0.019 (0.006)	0.98 (0.97, 0.99	< .001
Diagnosis of TMJ pain	No	0	1 (reference)	< .001
	Yes	0.559 (0.127)	1.75 (1.36, 2.46)	
Diagnosis of myofascial pain with referral	No	0	1 (reference)	< .001
	Yes	0.855 (0.117)	2.35 (1.88, 2.94	
Diagnosis of headache related to TMD pain	No	0	1 (reference)	< .001
	Yes	0.424 (0.117)	1.53 (1.22, 1.92)	
Degenerative joint disease—osteoarthritis	No	0	1 (reference)	.003
	Yes	0.447 (0.149)	1.56 (1.17, 2.09)	
Easy for patient to follow treatment recommendations	Ordinal	–0.154 .035)	0.86 (0.80, 0.92)	< .001
**Occlusal stabilization treatment** **^b^**				
In full-time practice	Part-time	0	1 (reference)	< .001
	Full-time	1.054 (0.262)	2.87 (1.72, 4.80)	
Years since dental school graduation	Interval	0.029 (0.007)	1.03 (1.02, 1.04)	.003
Expertise in TMDs and orofacial pain	No	0	1 (reference)	< .001
	Yes	–0589 (0.178)	0.55 (0.39, 0.79)	
Number of TMD patients seen in the past month	Interval	–0.35 (0.012)	0.97 (0.94, 0.98)	.003
Diagnosis of disc displacement with reduction	No	0	1 (reference)	< .001
	Yes	0.441 (0.147)	1.55 (1.17, 2.07)	
Ear pain	No	0	1 (reference)	< .001
	Yes	0.556 (0.153)	1.74 (1.29, 2.54)	
Change in occlusion	No	0	1 (reference)	< .001
	Yes	1.47 (0.151)	4.36 (3.25, 5.86)	
**Psychologic referral** **^c^**				
Female sex (practitioner)	Male	0	1 (reference)	< .001
	Female	0.942 (0.184)	2.57 (1.79, 3.68)	
Number of TMD patients seen in the past month	Interval	0.017 (0.005)	1.02 (1.01, 1.03)	.001
Number of dental patients seen per week	Interval	–0.023 (0.005)	0.98 (0.97, 0.99)	< .001
Diagnosis of myofascial pain with referral	No	0	1 (reference)	.002
	Yes	0.590 (0.191)	1.81 (1.24, 2.62)	
Diagnosis of headache related to TMD pain	No	0	1 (reference)	
	Yes	0.662 (0.206)	1.94 (1.29, 2.90)	.002
Diagnosis of degenerative joint disease—osteoarthritis	No	0	1 (reference)	< .001
	Yes	0.795 (0.215)	2.21 (1.45, 3.37)	
Easy for patient to follow treatment recommendations	Ordinal	–0.189 (0.042)	0.83 (0.76, 0.90)	< .001

*Values are adjusted for variables in the final model.

^a^Using listwise deletion, the final model for selected physical/biomedical treatments included 1,683 patients. Model fit: QICC baseline = 2,494.985; QICC final = 1,974.395.

^b^Using listwise deletion, the final model for selected occlusal stabilization treatments included 1,771 patients. Model fit: QICC baseline = 1,398.458, QICC final = 1,268.150.

^c^Using listwise deletion, the final model for a psychologic referral included 1,675 patients. Model fit: QICC baseline = 1,078.903, QICC final = 900.311.

Surgical and other treatment referrals were omitted because of their low frequency.

Once groups were formed based on each of the above seven treatment variables, the following sets of variables were tested as predictors of whether the practitioner made that treatment recommendation:

1. Dental practitioner/practice characteristics (gender; number of years since graduation; number of TMD patients seen in the past month; number of dental patients seen per week; expertise in TMDs and orofacial pain)
2. Practitioner expectation for outcome (relief from pain; improvement of jaw function; satisfied with treatment; treatment easy to follow; how well the patient understood the treatment recommendation)
3. Patient-related factors (patient gender, age, and insurance status; diagnosis of myalgia, ear pain, or headache related to TMDs; TMJ noise, limited jaw opening, and/or jaw stiffness or fatigue).

Statistical testing was performed using generalized estimating equations (GEEs). GEEs as used in this study are similar to binary logistic regression and allow the use of correlated data (SPSS version 26); in this report, the data were multiple diagnoses and treatment recommendations made by individual dentists. Testing for predictors of each treatment recommendation occurred progressively as follows:

- Block 1: Practitioner/practice-based variables and patient demographic and financial variables
- Block 2: Diagnosis
- Block 3: Symptoms
- Block 4: Practitioner assessment of expectations for outcome

Variables within each block that were significant at *P* < .01 were carried forward, and those that did not meet this critical value were removed prior to testing the subsequent block (ie, Block 1 to Block 2, Block 2 to Block 3). Patient gender, age, and insurance status were retained regardless of significance.

The final predictive models for each of the seven treatment categories were retested including only the variables that were retained in the final step. Binary predictor variables were coded as yes = 1 and no = 0 or as follows: male = 0, female = 1; part-time practice = 0, full-time practice = 1. Practitioner expectations for outcome (relief from pain, improvement of jaw function, satisfied with treatment, treatment easy to follow, and understood treatment) were scored on an 11-point scale anchored with 0 = not at all and 10 = complete relief/improvement or very satisfied/easy/well as appropriate. To evaluate the fit of the final model compared to the baseline patient demographic model, the corrected quasi-likelihood under independence model criterion (QICC) for each model was used.^16^ A lower value represents an improved model fit of the final model. The QICC is best used to test the fit between models rather than as an absolute measure of fit, as QICC values increase with sample size and autocorrelation; eg, the similarity of recommendations within practitioners.^17^

The unstandardized regression coefficient (B), standard error for B (SE), and odds ratio (OR) are reported. The coefficient B is interpreted similarly to the β value from ordinary least squares regression and is adjusted for the other variables in the final model. For every one-unit increase in the predictor (for interval variables) or factor (variables coded 0 or 1), B represents the direction and estimated change in the outcome. Similarly, the OR is the predicted change in odds with a unit change in the predictor variable. When the OR is > 1, increasing values of the variable correspond to increasing odds of the treatment being recommended. With an OR of < 1, increasing values of the predictor variable correspond to decreasing odds. For example, using the treatment recommendation intraoral appliance and the predictive factor years since graduation, the OR of 1.02 would be interpreted to reflect a 2% increase in recommending an appliance per year since graduation. Thus, a practitioner with 20 years since graduation would on average be 20% more likely to have recommended an appliance compared to those who graduated 10 years ago.

The data were then cleaned and recoded. Sets of treatment items (intraoral appliance, medications, self-care, additional treatments) were preceded by a single statement asking if any of that set of treatments was not recommended. If the “did not recommend” bubble was checked, all items in that set were scored as “not recommended.” If the “did not recommend” bubble was not checked, items within each set endorsed as yes were scored as positive, whereas items endorsed as no or a nonresponse within a set were coded as “not recommended.” A set of common TMD symptoms and potential diagnoses were also listed and scored in a similar manner. Consequently, the study analyses are based on positive or “yes” responses rather than positive vs true negative responses. Additionally, nonresponse was not recoded for items that have ordinal Likert-style responses, items that have multiple response options, demographic characteristics, and categories that do not correspond to yes/no.

## Results

### Participants

Of the 185 dental practitioners, 62% (n = 115) were male and 37% (n = 69) were female (1 did not reply). The majority graduated > 15 years ago from dental school (74%, n = 136), and 78% (n = 145) reported they worked full-time. Thirty-nine percent (n = 72) reported that one of their areas of expertise was TMDs and orofacial pain, with 24% (n = 44) reporting advanced training in an orofacial pain residency. Of the 1,901 patients who participated in the baseline evaluation, 83% (n = 1,584) were female and 17% (n = 316) were male. They were predominantly non-Hispanic and white (77%, n = 1,465). Characteristics of the study practitioners and their patients have already been described in Velly et al.^14^

### Diagnosis and Symptoms

The most frequently reported symptoms were jaw pain (91%), jaw stiffness or fatigue (74%), TMJ noise (64%), and headache (63%). The diagnoses given most often were myalgia (72%), headache related to TMD pain (51%), disc displacement with reduction (43%), and myofascial pain with referral (40%). A comprehensive list of diagnoses and symptoms reported for each patient in this study is available in the earlier publication.^14^

### Treatment Recommendations

#### Intraoral appliance

##### Frequency

An intraoral appliance was recommended to 1,434 (75%) patients.^14^

##### Predictors

As shown in Table 1, female patients and those with TMD insurance coverage were more likely to have an intraoral appliance recommended than male patients (*P* < .001) or those without insurance coverage (*P* = .002). Practitioners in full-time practice were more likely to include an appliance in the treatment plan than those who reported they were part-time (*P* < .001). In addition, a greater number of TMD patients seen in the past month and a greater number of years since dental school graduation were associated with a higher likelihood that the practitioner recommended an appliance (both *P* < .001).

Diagnoses associated with recommendation of an appliance were myalgia (*P* = .005) and headache related to TMD pain (*P* < .001). Patient symptoms associated were jaw stiffness or fatigue (*P* < .001). Practitioners who recommended an appliance were more likely to believe that the treatment would improve the patient’s jaw functioning (*P* < .001). Practitioner/practice- and patient-related factors predicting recommendation of an appliance are presented in Table 1.

#### Medications

##### Frequency

One or more medications were recommended to 1,094 patients (58%; see Table 5). The most frequently recommended medication was an over-the-counter (OTC) analgesic (44%).

**Table 5:** Medications for Treatment of TMD Pain Recommended by Study Practitioners

	% (n)
One or more medications recommended^a^	58% (n = 1,094)
OTC analgesics	44% (n = 828)
Prescription NSAIDS	16% (n = 305)
Muscle relaxant	14% (n = 268)
Other	5% (n = 86)
Tricyclic antidepressants	1% (n = 26)
Prescription narcotics	1% (n = 19)
Prescription cannabinoids	< 1% (n = 2)
No medications recommended	41% (n = 788)

Wording: What over-the-counter or prescription medication(s) did you recommend?

^a^50 dentists did not respond to any of the medication questions.

These data have been reported in an earlier manuscript.^14^

##### Predictors

As shown in Table 2, practitioners who reported their expertise included TMDs and orofacial pain were less likely to recommend a medication than those who did not report expertise in this area (*P* < .001).

The diagnosis of masticatory muscle pain with referral (*P* < .001) was associated with having one or more medications recommended. The patient symptoms associated were ear pain (*P* = .001) and jaw stiffness or fatigue (*P* < .001). Practitioners who recommended a medication were less likely to believe that the patient understood the treatment recommendations (*P* < .001).

#### Self-care and jaw exercises

##### Frequency

One or more forms of self-care behaviors were recommended to 89% of patients (n = 1,700; see Table 6). Jaw exercises were also recommended to 51% of patients (n = 938). 

**Table 6:** Patient-Initiated Care Recommended by Study Practitioners for Treatment of TMD Pain

	% (n)
One or more self-care activities recommended^a^	89 (1,700)
Avoid oral habits (eg, clenching or grinding teeth)	75 (1,418)
Relax your jaw muscles	73 (1,392)
Apply heat or ice	71 (1,343)
Eat a soft diet	70 (1,338)
Avoid chewing gum	69 (1,304)
Keep your teeth apart	57 (1,083)
Chew food on both sides	44 (838)
Reduce caffeine intake	27 (507)
Other	10 (182)
One or more jaw exercises recommended^b^	51 (938)
Self-massage of the jaw or temple	41 (773)
Jaw exercises	34 (644)

Wording:

Self-care: What self-care did you recommend?

Home jaw exercises: What additional treatment(s) did you recommend for their jaw or temple pain?

^a^51 practitioners did not respond to any of the self-care questions.

^b^56 practitioners did not respond to any questions on other treatments.

These data have been reported in an earlier manuscript.^14^

##### Predictors of self-care

Practitioner/practice- and patient-related factors as predictors of the recommendation of self-care and jaw exercises are presented in Table 3. Female practitioners were more likely to have recommended one or more forms of self-care than male practitioners (*P* < .001). Practitioners who reported their expertise was in TMDs and orofacial pain were more likely to recommend self-care than those who did not report this expertise (*P* = .006). In addition, the greater the number of years since dental school graduation (*P* = .005) and the greater the number of overall dental patients seen per week (*P* < .001), the higher the likelihood that the practitioner recommended self-care. However, a higher number of TMD patients seen in the past month was associated with a lower likelihood of recommending self-care (*P* < .001).

The only patient symptom associated with having self-care recommended was jaw pain (*P* = .004). Practitioners who recommended self-care were more likely to believe that the treatment would improve the patient’s ability to use their jaw (*P* = .009).

##### Predictors of practitioner-recommended jaw exercises

As shown in Table 3, female practitioners were more likely to recommend jaw exercises than male practitioners (*P* < .001). The diagnoses of myalgia (*P* < .001) and masticatory muscle pain with referral (*P* <. 001) were associated with a higher likelihood of having these exercises recommended.

The patient symptoms associated with having a jaw exercise recommended were TMJ noise, ear pain, and limited jaw opening (all at *P* < .001). Practitioners who recommended exercises as part of the treatment plan were less likely to believe the treatment recommendations would be easy to follow (*P* < .001).

#### Referral to specialized health care providers

##### Frequency

Practitioners also referred their TMD patients for additional treatments, including referral for selected physical/biomedical treatments (40%), occlusal stabilization treatment (15%), and psychologic counseling (8%).

##### Predictors of selected physical/biomedical treatments

Practitioner/practice- and patient-related factors predicting practitioner recommendations for physical/biomedical, occlusal adjustment, and psychologic treatments are presented in Table 6. Practitioners who were female and those who reported their expertise was in TMDs and orofacial pain (*P* = .001) were more likely to recommend referral for physical/biomedical treatment than their counterparts (*P* < .001). A greater number of TMD patients seen in the past month (*P* < .001) was associated with a higher likelihood of referral for physical/biomedical treatment. However, a greater number of overall dental patients seen per week (*P* = .001) was associated with a lower likelihood of referral for physical/biomedical treatment.

The diagnoses of TMJ pain (*P* < .001), myofascial pain with referral (*P* < .001), headache related to TMD pain (*P* < .001), and degenerative disc disease/osteoarthritis (*P* = .003) were associated with the recommendation for physical/biomedical treatment. Practitioners who recommended physical/biomedical treatments were less likely to believe the treatment recommendations would be easy to follow (*P* < .001).

##### Predictors of occlusal stabilization treatments

Practitioners in full-time practice and those who did not report their expertise was in TMDs and orofacial pain were more likely to recommend occlusal stabilization treatments than practitioners who reported they worked part-time or those who did not report this expertise (both *P* < .001; see Table 6). In addition, a greater number of years since dental school graduation and fewer TMD patients seen each month were both associated with a higher likelihood that the practitioner recommended occlusal stabilization treatments (both *P* < .003). 

The diagnosis of disc displacement with reduction was associated with referral for occlusal stabilization treatments (*P* < .001). The patient symptoms associated were ear pain and change in occlusion (both *P* < .001).

##### Predictors of psychologic treatment

Practitioners who were female were more likely to have recommended a referral for psychologic treatment than male practitioners (*P* < .001; see Table 6). In addition, the greater the number of TMD patients seen in the past month and the fewer the number of overall dental patients seen per week, the greater the likelihood of a referral for psychologic treatment (both at *P* < .001).

The diagnoses of myofascial pain with referral (*P* = .002), headache related to TMD pain (*P* = .002), and degenerative joint disease/osteoarthritis (*P* < .001) were associated with referral for psychologic treatment. Practitioners who recommended psychologic treatment as part of the treatment plan were unlikely to believe that the treatment plan would be easy to follow (*P* < .001).

## Discussion

This study found that dental practitioners often recommended multiple treatments and usually provided several TMD diagnoses for each patient. Practitioners generally used a triad of recommendations that consisted of self-care, medications, and intraoral appliances. They primarily focused on TMD signs and symptoms when making treatment recommendations. 

### Intraoral Appliances

One of the most common treatment options for individuals experiencing pain-related TMDs are removable intraoral appliances.^1,18–25^ In this Network sample, the recommendation rate for an appliance was 75%.^14^ Female patients were more likely to have been recommended an appliance than male patients. Although women are known to be at increased risk for experiencing pain-related TMDs,^21^ we are unable to find studies that examined sex differences for treatments recommended. To examine this apparent bias, post hoc analysis showed no sex differences in specific diagnoses or symptoms that were significantly associated with an appliance recommendation. However, the subset of female patients received a greater number of overall diagnoses and had more identified symptoms than their male counterparts. There were also no sex differences in practitioner expectations for outcome among patients who were recommended an appliance. In addition, insurance coverage was associated with the recommendation for an appliance. This association may be explained by the cost of appliances, as they can be expensive.^22,23^

An appliance was the most frequently recommended single treatment, especially among practitioners with a greater number of years since dental school graduation. This may be a function of younger practitioners who often choose to refer complicated cases to more experienced colleagues.^26^ Reviews have reported evidence for the short-term effectiveness of an appliance in reducing pain-related TMDs, particularly that of muscular origin,^10,19^ although many studies lack strong experimental designs.^25^ This is supported by supplementary analysis of the 87% of patients recommended an appliance, which revealed that the primary goal of this recommendation was muscle relaxation, followed by unloading the joint and pain relief (87%, 67%, and 55%, respectively). Correcting malocclusion was reported for only 18% of patients. The high use of appliances with limited evidence of effectiveness could be partially explained by alignment with the practitioner’s training and skills, the desire to provide an active treatment that could benefit the patient, or because providing an appliance can be profitable.

### Medication

Pharmacotherapy is typically used to target somatic symptoms associated with pain-related TMDs such as inflammatory diseases, chronic pain, arthralgias, or myalgias.^26,27^ In the current study, recommendation of a medication was most common with muscle pain disorders. OTC analgesics were the most common and often the only medication recommended. Prescription NSAIDs were the next most common prescribed medication, although our study did not identify the type of prescription NSAIDs recommended. Narcotic prescriptions were seldom provided. Reporting TMDs and orofacial pain as an area of expertise was associated with not recommending a medication, despite these practitioners treating more complex cases (ie, patients with a greater number of TMD diagnoses and symptoms). This suggests that providers with expertise in TMDs may have preferred other treatment modalities over medications. A previous meta-analysis suggested mixed results for use of pharmacologic treatment for pain-related TMDs.^28^

### Patient-Initiated Care

Self-management of TMD symptoms consists of a wide range of different self-care treatments and exercises.^29^ Many of the self-care recommendations are meant to increase cognitive awareness of oral parafunctional behaviors.^27^ Others involve adaptation at meals and/or target pain.^30,31^ We found that these simple noninvasive behavioral strategies were recommended to nearly every patient, and typically three to five were recommended at a time. Avoiding behaviors that involved clenching and grinding of the teeth were most often suggested, as well as relaxing the jaw muscles. Female practitioners were more than three times as likely to recommend self-management strategies as male practitioners, consistent with the conservative approach taken for other preventive and conservative treatment strategies.^11,12^

Just over half the patients were recommended at least one jaw exercise, and more often than not, multiple exercises were recommended together. Although we asked about exercises recommended by the treating practitioner, they could be part of a subsequent treatment plan provided by a physical therapist or massage therapist for patients to do on their own.

Several studies have examined the effectiveness of a wide range of self-management and exercise programs, often in comparison with other treatments.^32,33^ For example, one study found that jaw exercises had positive outcomes for pain and joint sounds that were similar to patients receiving an intraoral appliance.^34^ Santiago and Raphael^23^ found that patients with myofascial TMDs reported improvement was the highest with self-management activities, suggesting that self-care measures should be part of treatment plans for this set of patients.

### Recommendation for Additional Treatment

Over 80% of the pain-related TMD patients were recommended one or more of the specialized treatments. We divided these into three categories: physical/biomedical, occlusal, and psychologic counseling.

#### Physical/biomedical treatment referrals

Of the physical/biomedical treatment referrals, the most common was physical therapy, followed by massage therapy. Physical therapy focuses on reduction of pain and improvement in jaw function. Physical therapy is effective in reducing pain-related TMDs^35–37^ and may work best as part of a multidisciplinary program.^6^ Massage therapy may address myofascial pain that results from clenching of the teeth and stress, while reestablishing the proper flexibility and muscular length may also relieve pain.^38^ Massage therapy has also shown positive outcomes.^39,40^ Chiropractic care^41–43^ and trigger point injections^44^ were seldom included in the treatment plan. For this set of referrals, female practitioners and those with expertise in TMDs were more likely to refer to allied health care providers. Given the apparent effectiveness of physical and massage therapy, the low referral rate suggests underutilization.

#### Occlusal stabilization treatment

Occlusal stabilization treatments were recommended to 15% of the patients and included occlusal adjustments, orthodontics, restorative treatment, and full-mouth reconstruction. Network practitioners may have intended to perform these procedures or refer to a more specialized practitioner. The frequency of occlusal adjustments reported in this study is considerably lower than the 64% reported in an earlier survey of dental practitioners for the treatment of TMDs.^5^ A concern has been raised about the cause-and-effect relationship whereby occlusal changes are often secondary to TMDs.^6,45^ There is little evidence that occlusal treatment results in positive long-term outcomes.^45,46^ Of the diagnoses and symptoms, the patient reporting a change in occlusion was the strongest predictor of the likelihood of recommendation of this treatment, suggesting that this treatment may have been warranted as a secondary treatment if this finding did not resolve with nonocclusive primary treatment. Full-time practitioners were more likely to recommend both appliances and irreversible occlusal treatments compared to part-time practitioners. Our data do not allow us to determine if the appliance was used to test for occlusal factors in the etiology of TMDs. Nevertheless, full-time practitioners recommended both an appliance and occlusal treatments to 15% of patients, compared to only 4% for part-timers. What we can say is that full-time practitioners treated fewer TMD patients and were less likely to report expertise in TMDs but see a greater overall number of dental patients in their practice. 

#### Psychologic counseling

It is commonly acknowledged that psychologic factors, including stress and anxiety, play a role in the development and maintenance of TMDs.^34,47^ Consequently, therapies involving relaxation, stress management through cognitive behavioral therapy, and education can be helpful.^48^ Biobehavioral treatments can increase pain control, improve negative affectivity associated with TMDs, and may also target maladaptive oral behaviors.^49^ In this sample, we found that referral for psychologic treatment was infrequent. The lack of consideration of psychosocial aspects of TMDs has been reported as a common barrier in management of pain-related TMDs.^50^ It is possible that psychosocial factors are not sufficiently recognized by practitioners as a risk for onset and progression of TMDs, particularly complex cases^6^ or when there is a lack of access to a pain psychologist who can help the patient understand and address this relationship. Also, practitioners may not be comfortable assessing psychosocial factors. Female practitioners were more than twice as likely to recommend psychologic treatments compared to male practitioners. Degenerative joint disease/osteoarthritis and joint pain were associated with increased likelihood of referral for treatment, suggesting that practitioners perceive that learning these skills will better equip the patient to manage a chronic joint condition.

### Practitioner Expectations of Patient Outcomes

Practitioner expectations were not strong factors in predicting treatment choices, although there were exceptions. Several earlier studies have suggested that health care providers are poor judges of patient viewpoints.^51,52^ The recommendations for an intraoral appliance or self-care were positively associated with expectations for improvement in jaw function, and these treatments were the most often recommended. Conversely, practitioners expected patients to have difficulty with the treatment plan when they referred them for physical/biomedical or psychologic counseling. Practitioners were potentially concerned about compliance with more complicated treatment plans. This suggests that practitioners should provide their patients with rationale for these referrals to improve compliance.

## Conclusions

Practitioners primarily considered TMD signs, symptoms, and diagnoses when making treatment recommendations, suggesting a focus on the biomedical model. One challenge with TMD pain is determining which factors have prognostic significance and aligning these factors with appropriate treatment recommendations. Consistent with the complexity of TMDs, the biopsychosocial model advances our understanding of pain-related TMDs.^46^ Practitioners with expertise in TMDs and those who treat greater numbers of TMD patients were most likely to refer patients for specialized treatment. This supports advanced training within residency programs or evidence-based TMD continuing education seminars to teach practitioners about the complexity and multicausality typical of TMDs.^48,50,53^ Female practitioners and practitioners treating more pain-related TMD patients are more likely to develop referral relationships with providers in other disciplines. Results from this study suggest both groups prefer patient-directed, multidisciplinary treatment recommendations. 

A limitation of this study is that the analyses are based on positive or “yes” responses vs not positive or “not yes” responses rather than on positive vs true negative responses, as this could cause misclassification. In addition, the results may not generalize to non-Network dental practitioners. Although we had a large sample of patients and practitioners across all regions of the Network, selection bias is likely to have occurred with practitioners who chose to enroll in this study, as well as from the patient’s choice of dental practitioner and practice setting. Multiple other variables may be important predictors of treatment decision for TMDs that were not included in our analysis and would have improved the fit of our models.

Overall, TMD patients can be challenging to treat, and the need for additional training in dental schools has long been recognized.^54,55^ However, dental practitioners receive limited training in TMD diagnosis and management in dental school.^54^ Treatment concepts are changing from dental-based biomedical causes to the complexities of the biopsychosocial model. More than ever, evidence-based training in dental schools is needed to teach students how to assess and treat patients with simple presentations and when to refer complex patients to a specialist for other services.^56,57^ In addition, it is suggested that practitioners consider continuing education courses on TMDs and orofacial pain that include seminars that present the biopsychosocial perspective.

## Highlights

- Practitioners primarily used a triad of recom-mendations that consisted of medications, an intraoral appliance, and patient self-management.
- TMD signs and symptoms were strong predictors of the treatment plan recommended, whereas the practitioner’s expectations for improvement were only significant for self-care and an intraoral appliance.
